# Measuring Theory of Mind in Adolescents With Language and Communication Problems: An Ecological Perspective

**DOI:** 10.3389/fpsyg.2022.761434

**Published:** 2022-04-25

**Authors:** Lidy Smit, Harry Knoors, Inge Rabeling-Keus, Ludo Verhoeven, Constance Vissers

**Affiliations:** ^1^Royal Dutch Kentalis, Sint-Michielsgestel, Netherlands; ^2^Behavioral Science Institute, Radboud University Nijmegen, Nijmegen, Netherlands

**Keywords:** theory of mind (ToM), adolescents, developmental language disorder (DLD), deaf and hard of hearing (DHH), ToMotion, ToM task

## Abstract

We tested if the newly designed ToMotion task reflects a single construct and if the atypical groups differ in their performance compared to typically developing peers. Furthermore, we were interested if ToMotion maps a developmental sequence in a Theory of Mind (ToM) performance as exemplified by increasing difficulty of the questions asked in every item. The sample consisted of 13 adolescents that have been diagnosed with a developmental language disorder (DLD) and 14 adolescents that are deaf or hard of hearing (DHH). All of these adolescents were in special schools for secondary vocational education. The control group existed of 34 typical developing adolescents (TD) who were in regular intermediate vocational education, ranging from level 2 to 4. The ToMotion, available in a spoken Dutch version and in a version in Sign Language of the Netherlands (SLN), was used to map ToM abilities. An attempt has been made to fill the gap of missing studies of ToM in adolescents by developing a new measuring instrument. In conclusion, assessing ToM with the ToMotion results in a picture that DHH adolescents score lower than TD peers. However, their scores are as consistent as those of the TD peers. The picture of DLD adolescents is the reverse. They show no differences in ToM scores, but seem to be somewhat more inconsistent compared to TD peers. We provide a discussion on those results and its implications for future research. What this paper adds? The current study introduces a new visual Theory of Mind (ToM) task, ToMotion, designed specifically to assess ToM in adolescents in an ecologically valid way and adapted to the needs of adolescents with language and communication difficulties.

## Introduction

Research into social cognitive processes, such as Theory of Mind (ToM), maps the ability of human beings trying to understand themselves and others around them.

Theory of Mind development is closely intertwined with language development (Milligan et al., 2007; Ebert, 2020; Richardson et al., 2020). Longitudinal studies show that communication about the mental representations of the inner world contributes to the proper development of ToM (e.g., Lohmann and Tomasello, 2003; Milligan et al., 2007; Nilsson and de López, 2016; Ebert et al., 2017; Ebert, 2020). Language skills give better access to another person’s mental states, as language is a medium to represent mental states. Verbal interaction enables a person to learn more about their thoughts, intentions, and feelings (Saxe and Baron-Cohen, 2007). With that in mind, studies of ToM performance seem especially relevant for two groups of children known to be at risk for language and/or communication problems; that is, children with Developmental Language Disorder (DLD) or those who are Deaf or Hard of Hearing (DHH). Reduced access to language (both inner speech and communication skills to the outside world) hinder the mentalization of one’s own and others’ inner worlds (ToM) (e.g., Vissers and Hermans, 2018; Camminga et al., 2021).

Although there are many studies that assess ToM in children, to the best of our knowledge, there is a relative lack of studies that address ToM in adolescents, especially in adolescents with language and communication problems (Smit et al., 2019). One of the causes might be that ToM tasks suitable to assess ToM in children are not suitable for adolescents and adults due to ceiling effects. Furthermore, ToM tasks for children are not appealing to adolescents, rendering them ecologically invalid. The first ones to try to overcome these problems through developing ecologically valid test materials were Murray et al. (2017) with the strange stories film task (SSFT) that was specifically aimed at assessment of ToM in adults. However, the task they have developed is strongly focused on the living environment of adults. For example, scenes about cooking dinner or visiting a dating site. In addition, the language of the task has limited access for adolescents with language and communication problems. Therefore, we developed a new diagnostic tool from an ecological valid perspective that respects the natural ToM sequence in real-life situations and is accessible to adolescents with and without communication and language problems in order to map their ToM ability.

### Theory of Mind From a Developmental Perspective

Theory of mind is an abstract representation of one’s own and others’ mental states. Based on these mental representations, we can assess the behavior of ourselves and others (Perner, 1991). ToM is indispensable for the proper adjustment of our own behavior in social situations and is necessary to avoid misunderstandings in social exchanges. The ToM construct can be differentiated into two subcomponents: cognitive theory of mind, which describes a cognitive knowledge about beliefs, and affective theory of mind, which describes knowledge about emotions (Westby and Robinson, 2014).

In real-life situations, the formation of a ToM proceeds in a sequence. The first step in forming a ToM is focusing one’s attention on a social situation and perceiving the cues that are important to assess the situation (Dodge et al., 1986; Baron-Cohen, 1991; Crick and Dodge, 1994). In the second step, understanding of intentionality must arise; that is, the understanding that actions of others are goal-directed and arise out of unique beliefs and desires (Dennett, 1983). Subsequently, one has to establish physical and psychological presence and adjust the body and mind to the people around someone (Crick and Dodge, 1994). This sequence places ToM development parallel from childhood.

The development of ToM, which ultimately enables someone to adequately function in increasingly complex social exchanges, starts during childhood. The foundation for ToM is already laid at birth (Keysers, 2011; Andreou and Skrimpa, 2020), where mirror neurons contribute to the ability to live through the emotions of another. About the age of 6 months old, a child shows the first emotions, i.e., joy, sorrow, disgust, anger. Also, the child follows the gaze of another person, the so-called “joint-attention,” which is the first step in learning to focus on cues that are important for social interaction (Tomasello, 1995; Westby and Robinson, 2014). From 8 to 12 months old, the child begins to understand that there is a relationship between a person’s sight and their perception (Westby and Robinson, 2014). From 18 months to 4 years old, children increasingly realize that they are an individual that is separate from others and experience having different desires and needs than others, which is an important realization to form a ToM. The child begins to display affective empathy and altruism, by, for example, helping or comforting others (Thompson and Newton, 2013). Children begin pretend play and playfully develop the ability to consciously reflect on their physical appearance and behavior (Perner, 1993). Through play and experience, children become acquainted with the limits of their physical condition, nourishing their inner world and self-experience. Consequently, children start to use words that are emotionally related, such as happy, afraid, angry, and sad. Their spontaneous speech contains words such as thinking and knowing. Parallel to these behavioral changes, in the brain, we see the emergence of networks involved in reasoning about mental states (Richardson et al., 2018). Around 4–5 years old, the ability to form a first-order ToM develops. Children can then form a simple mental representation of what another thinks or feels. In this phase, the child can recognize other persons’ false beliefs about reality. Children develop the ability to recognize and associate emotions (happy, scared, sad, angry, surprised, disgusted) with specific events (e.g., “Noah is angry because he is not allowed to ride a bicycle”; Michalson and Lewis, 1985; Pons et al., 2004). By communicating about emotions, children gain insight into cause and effect, intentions, and consequences, and language bridges the gap between a child and the other. Most children gradually acquire this insight as they occasionally find themselves in a variety of social situations through everyday life. As a result, children develop a variety of mental words (for example: think, know, want, wish) (see Stanzione and Schick, 2014). Immediately after the development of a first order ToM, between 6 and 12 years of age, they develop the ability to predict not only what someone else thinks or feels, but also what another person thinks of how someone else feels or thinks (e.g., “Noah hopes that his sister Amy believes she knows what their mother wants for her birthday”). This ability is called the second-order ToM (Miller, 2009; Im-Bolter et al., 2016), where the understanding of lies, sarcasm, and imagery or faux-pas (something said unintentionally hurts the other) emerges.

There is a gap in research about what happens after the development of the second-order ToM (at around the age of 12 years) (see Wiley et al., 2020). It is precisely during this phase that the transitional phase of growth and development between childhood and adulthood, ranging from 13 to 23 years, when ToM skills are indispensable to fit in the social world. The primary challenge of adolescence involves the formation of new social relationships that results in feelings that one matters and that one is respected in order to make a valued contribution to society (Dahl et al., 2018). From a neural perspective, there is evidence that brain areas used for reasoning mental states become more specialized throughout middle childhood into early adolescence (Lagattuta et al., 2015). This process is related to ToM abilities (Moriguchi et al., 2007). Findings regarding ToM performance during adolescence are inconsistent (see for an overview Gabriel et al., 2021). Age−related improvements from late adolescence into adulthood appear to vary depending on the type of ToM measures used (Tousignant et al., 2017). The lack of ecological valid instruments to assess ToM functioning in adolescents and adults may be an important reason for the relative lack of knowledge about the development of ToM during adolescence. Quantifying ToM levels in adolescents is a challenge. ToM in children is often assessed through “false belief” tasks that require first−order [e.g., “what does Sally (mistakenly) think”] and, later, second−order mental state attribution [e.g., “what does John (mistakenly) think that Mary thinks”; Baron-Cohen et al., 1985; Baron-Cohen, 1989; Happé, 1994]. ToM has also been assessed using the Strange Stories task, wherein the participant has to interpret non−literal statements as a lie, joke, or bluff in the context of social narratives (Happé, 1994). However, ceiling effects are often observed on such tasks (which developing 5–7−year−olds typically pass). The written format of strange stories and false belief tasks ignores the ability to process natural non-verbal and verbal cues, as is the case in everyday social situations. Murray et al. (2017) tried to overcome the lack of ecological test material to measure ToM in English speaking adults with the strange stories film task (SSFT). Several videoclips were made based on the scenarios from the Strange Stories task (Happé, 1994). The SSFT uses naturalistic cues in video format. Unfortunately, due to the language used and the content of the scenarios, the SSFT is not suitable for Dutch speaking adolescents. The content of the task is strongly aimed at adults, which makes it difficult for adolescents to relate to. In addition, a version in sign language would be an added value in order to make the task accessible for DHH adolescents. Three factors were used in the SSFT to assess social understanding following the viewing of a clip: (a) Intention, (b) Interaction, and (c) Memory Question. However, if we look at ToM development from childhood, in our opinion, there lacks an important question that maps whether the participant has detected the correct social cues in the clip and is able to explicitly appoint them. As a result, information is lacking on the way in which a participant forms his final ToM about the situation.

### Theory of Mind in Adolescents With Language and Communication Problems

Adolescents with DLDs and adolescents who are DHH are at greater risk of social emotional problems (Smit et al., 2019).

Interactions between language development, cognitive development (as is ToM), and social emotional problems have been studied in children with DLD and in DHH children (e.g., Nilsson and de López, 2016; Vissers and Koolen, 2016; Walker et al., 2017; Peterson and Wellman, 2018), but a possible interaction between these factors has not been studied in adolescents. In our opinion, there is a serious omission, because adolescence is a time of considerable development at the level of behavior, cognition, and the brain (Blakemore and Choudhury, 2006). It is important to shed light on the interaction between these factors to develop intervention programs to tackle social and emotional problems as seen in adolescents with DLD and those who are DHH (Smit et al., 2019).

For DHH children, research shows that ToM is delayed in DHH children with hearing parents (Walker et al., 2017; Peterson and Wellman, 2018) and not in those with deaf parents (i.e., native signers; Schick et al., 2007). This underlines the importance of a shared and accessible language for ToM development. In individuals with DLD, the cause of the language problems is different from DHH children. The roots of the language problems are at the neurocognitive level where language is not properly processed. For children with DLD, research demonstrates deficits in ToM, which is already evident in preschoolers with DLD (Vissers and Koolen, 2016). Vissers and Koolen (2016) proposed three models to explain the interplay between ToM, language, and social-emotional functioning. In the first model, ToM facilitates language development. The second model states that language stimulates the development of ToM. The third model suggests that language and ToM deficits coexist because they are fueled by a single neuropsychological underlying structure, such as working memory (WM), an aspect of executive functioning.

There are few studies that focus on ToM functioning in adolescents with DLD or are DHH (Smit et al., 2019). For typically developed adolescents, adolescence is a turbulent phase. Hence, it must be even harder for the atypical groups. As far as our knowledge reaches, there is only one study that looks at ToM skills in adolescents with DLD. Clegg et al. (2005) performed a cohort study in which 17 men with DLD in childhood (4–9 years) were reassessed in middle childhood and early adult life (early 20s), and again in their mid-30s. It revealed that these men scored lower than their TD peers and siblings on three ToM tasks, namely, strange stories task, awkward moments task, and reading the eyes in the mind task. Studies on ToM performance in DHH adolescents are limited in number. Most research has focused on false belief understanding in DHH adolescents using false belief tasks. Russell et al. (1998) found significant improvement on first-order false belief reasoning (using the false belief task) at the age of 13–16 years. Using the Look Prediction Task, the Strange Stories task, and the Reading The Mind in The Eyes Test, Lecciso et al. (2016) found that late signers and oral deaf adults (age range: 15–28 years) scored lower than their TD peers. Marschark et al. (2018) assessed ToM performance in 94 deaf adolescents attending university by evaluating their understanding of sarcasm and advanced false belief (second-order false belief and double bluff). Consistent with the previous studies, deaf participants scored lower than hearing peers. de Gracia et al. (2020) performed the first study on ToM performance beyond false belief understanding in a non-Western deaf population (age range: 15–22 years) using the ToM scale (Wellman and Liu, 2004). Deaf adolescents displayed significantly lower ToM scores compared to the younger 8–14-year old hearing participants. Figueroa et al. (2020) studied 36 adolescents (12–16 years of age) with a cochlear implant (CI) on reading and ToM, and results indicated that reading and cognitive ToM were more developed in the TD group than in adolescents with a CI. However, in all the aforementioned studies, the ToM tasks did not display naturalistic cues. Neither did they map the ToM sequence in social situations in an ecologically valid way.

### Present Study

The current study introduces a new ToM task, ToMotion, that was specifically designed to assess cognitive ToM in adolescents in an ecologically valid way while concurrently adapted to the needs of adolescents with language and communication difficulties. ToMotion tries to measure the cognitive dimension of the ToM construct through different social situations (items) that are recognizable to adolescents. We suggest that ecologically valid tasks assessing ToM take in account the following: (a) Using dynamic stimuli, (b) showing real persons, and (c) displaying everyday life situations to ensure the possibility of using contextual information to draw conclusions (Murray et al., 2017; Feyerabend et al., 2018). Content-wise, it is important to mirror real-life sequences in situations with an developmental perspective on ToM (Crick and Dodge, 1994). Particularly, (1) Is the person able to focus his attention on the context of the situation and extract the important elements from it?; (2) Is the person able to understand what intentions, beliefs and desires are involved in this situation?; and (3) Is the person able to tune in to the situation, taking into account the above elements, and give an appropriate response? The above questions follow the natural ToM development as it takes place in childhood, namely, from the joint attention (Tomasello, 1995; Westby and Robinson, 2014) to the first-order ToM (4–5 years of age), where the child can form a simple mental representation of what another thinks or feels (Michalson and Lewis, 1985; Pons et al., 2004); to the second order ToM (6–12 years of age), where the ability to predict what someone else thinks or feels emerges, along with what another person thinks someone else feels or thinks; and in coming up with an appropriate response (Miller, 2009; Im-Bolter et al., 2016). Test conductors gain insight into the extent to which a test subject is able to understand and respond according to the ToM sequence in various social vignettes, and whether the subject can consistently endure the sequence across all items. We have taken all the above aspects into account in the design of the ToMotion. The tasks mainly involve thinking about the thoughts, knowledge, beliefs, and the intentions of others, which falls under the sub component cognitive ToM (Westby and Robinson, 2014). The sub-dimension, affective ToM, which can be described as the ability to respond to the emotions of others (i.e., to feel as others are feeling), is not explicitly measured in this task.

If the items indeed predominantly measure cognitive ToM, one would expect all items to reflect a single construct. Hence, the first research question is formed as follows: do the 12 items together reflect a single construct?

We expect that both the DHH adolescents and the adolescents with DLD exhibit lower levels of ToM performance compared to typically developing peers. Hence, the second research question is as follows: Do the atypical groups differ in their performance on the ToMotion compared to typically developing peers?

The third research question is as follows: does the ToMotion map a developmental sequence in ToM performance as exemplified by increasing difficulty of the questions asked in every item? In addition, are there differences in this sequence, both in terms of order as in terms of consistency between typical and atypical groups?

From a developmental perspective, we hypothesize that question 2 is more difficult than question 1 and that question 3 is more difficult than question 2. Furthermore, we hypothesize that the typical groups show more consistency in the ToM sequence than atypical groups.

## Materials and Methods

### Construction of the ToMotion

The ToMotion is a test of ToM that is available in a spoken Dutch version and in a version in Sign Language of the Netherlands (SLN). ToMotion consists of video clips that portray scenarios of everyday-life social situations. These scenarios were derived from Happé’s Strange Stories task (Happé, 1994) and include lie, white-lie, joke, pretense, idioms, persuasion, appearance/reality, misunderstanding, forgetting, double bluff, contrary emotions, and irony. For a screenshot of the spoken version and the SLN version see pictures below.

In order to make the task ecologically valid, in creating the clips, the everyday environment of young people has been taken into account. The clips were filmed in, for example, a canteen at school, schoolyard, school corridor, near a coffee machine, or near school lockers. To make the task accessible to both hearing adolescents with DLD and DHH adolescents, the language that was used in the clip differed: spoken Dutch (with adaptations) for adolescents with DLD, and SLN for DHH adolescents. The language used is focused on everyday conversations between young adolescents.

In the spoken version, Dutch utterances were adapted for adolescents with DLD. All utterances are relatively short and to the point (see Supplementary Appendix 1 for a description of the scripts used). Actors for the spoken version were young adults who were fluent in Dutch. Both adults have affinity with acting, but they were not professional actors.

For the sign language version, consultation took place with the Dutch Sign Language Center. The Dutch Sign Language Center is the national expert center for SLN. It was checked whether the signs used in the ToMotion match the signs used by deaf young people in the Netherlands. The actors in the sign language version were both native signers.

**FIGURE 1:**
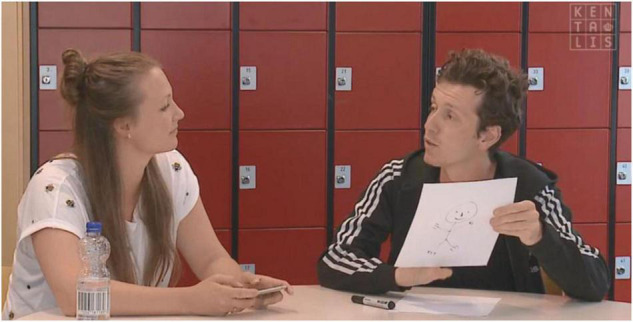
Screenshot of the clip “white lie” in Dutch Spoken Language.

**FIGURE 2:**
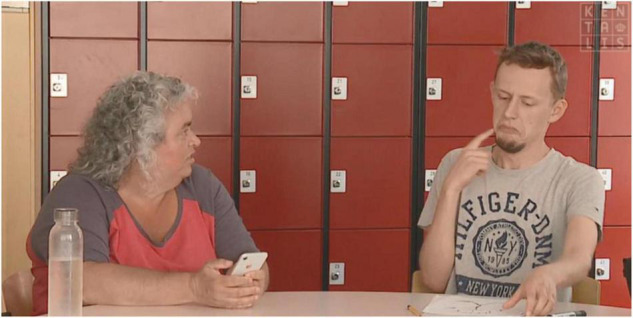
Screenshot of the clip “white lie” in Dutch Sign Language.

During filming, care was taken to ensure that emotional expression on the face was clearly visible and that body posture and context are clearly portrayed, just like in real life conversations. The clips were filmed by a professional camera team with professional equipment (camera: Sony, Japan, EX-1, microphone: Sennheiser, Germany, MKH-30) to guarantee high video quality and clear sound. In total, 13 clips were filmed, namely, 12 test items and 1 clip to practice at the beginning (for a complete overview of all clips used, see Supplementary Appendix 1). The clips in the spoken version and the clips in the sign language version were identical, except for the language being used. See Figure 1 for an example from the spoken version and Figure 2 for an example for the sign language version.

#### Questions

To assess social understanding, after the viewing of each clip, the following questions were asked:

(1) A descriptive question: “What did you just see happen?”
(2) An explanatory question: “Why did X say this?” (referred to the last speaker).
(3) A personalized question: “What would you do if you were Y?”

Questions 2 and 3 were also used by Happé (1994) in the strange stories task. Question 1 was added in the ToMotion to better map the real-life sequence by not ignoring drawing attention to the social situation—the essential first step.

These questions were asked by a psychologist in case of the DHH group, with support of a qualified SLN interpreter. The reaction of the participant to question 1 reflects what they are thinking after seeing a social situation without the test leader directing it in a certain way. It is examined whether the participant detects elements from the social context and whether they can name them. This gives us insight into the linguistic social information processing and whether the participant pays attention to social cues, parallel to the first step required to form a ToM in a real-life social situation. The second question tests to what extent the participant understands intentionality and to what extent he/she understands that others’ actions are goal-directed and arise out of unique beliefs and desires. The third question calls on the internalization of the above information. The answer on this question gives insight in the ability of the participant to attune in a social, communicative situation and give a relevant response. The personalized question asked about a possible response to the final utterance of the clip: “If you were in Y’s (other character i.e., not X) situation, what would you say next?”

#### Scoring

There is a varying level of difficulty in the questions, with respect to applying ToM in everyday situations and with respect to the developmental perspective on ToM. For a proper application, it is necessary to first pay attention to the social cues (question 1), to take in account the intentions of the other (question 2), and then adjust your communication and behavior to the social context and people around you (question 3). Depending on the accuracy of the answers given by the participants, each question was scored with 0, 1, or 2 points, with 0 reflecting the lowest and 2 the possible highest level of social understanding and acting. In order to calculate the overall attained level of ToM, the scores on the descriptive question (1), explanatory question (2), and personalized question (3) were added up for each ToM item. A ToM item thus has a total score range of 0–6. A reflection of a good ToM is shown by a consistently high score of 2-2-2 on an item, resulting in a total score of 6 on one item. Then, the ToM total score adds up the score on the 12 ToM items with a range of 0–72. A higher ToM total score is an indication of a higher ToM competence if it is supported by a high consistency score.

In Supplementary Appendix 2, the script of the video fragment “idiom” and the corresponding scoring template can be found.

In addition, each of the 12 ToM items was categorized as showing either a consistent pattern (C) or an inconsistent pattern (I). In Supplementary Appendix 3, the format for scoring consistency and the frequency per item can be found. This categorization was based on the answers given on the 3 questions of each ToM item. Particularly, consistent patterns reflect scoring patterns that run parallel to the difficulty within the items.

For example: 2-1-1. Inconsistent patterns do not reflect the increasing difficulty within the items; for example a pattern like 0-0-2. The participant eventually gets a 1 for a consistent pattern or a 0 for inconsistent pattern for the entire item.

### Participants

Data from 61 participants were analyzed (see Table 1 for demographic details). The sample consisted of 13 adolescents that had been diagnosed with DLD and 14 adolescents that were DHH. In the DHH sample, 3 adolescents had a deaf parent, while 11 had hearing parents. All adolescents were in special schools for secondary vocational education. The control group consisted of 34 adolescents who were in regular intermediate vocational education, ranging from level 2 to 4. Informed consent was obtained from the participants and their parents/caregivers. There was no reward for participation.

**TABLE 1:** General characteristics of the study sample, namely, children in the TD-group (*n* = 34), children in the DLD group (*n* = 13), and children in the DHH-group (*n* = 14), expressed in absolute numbers (percentages between brackets) or in means (standard errors between brackets).

Characteristics	TD-group (*n* = 34)	DLD–group (*n* = 13)	DHH-group (*n* = 14)	Test statistic and *p*-value
Age				*F*(2, 58) = 15.727, *p* < 0.001*
Mean (SD)	18.0 (0.29)	15.7 (0.47)	15.5 (0.45)	
Sex				*X*^2^(2) = 0.296, *p* = 0.862
Female (%)	16 (47.1%)	5 (38.5%)	6 (42.9%)	
Male (%)	18 (52.9%)	8 (61.5%)	8 (57.1%)	

One-way ANOVA and Chi-square tests were performed to determine whether the groups were significantly different from each other on the characteristics. *Pairwise comparisons showed that the mean age in the typical developing adolescent (TD) groups is significantly higher than the mean age of the other two groups (both p-values < 0.001). The mean ages of the two other groups do not significantly differ from each other p = 0.768).

### Procedure

For all participants, testing took place in a small and quiet room at school with little to no distraction. Participants were seen individually, and the testing took up to an average of 30 min. All answers of the DLD group are recorded with audio recorders (Olympus LS-P1) and later transcribed. During the testing of the DHH group, a qualified SLN interpreter was present. In the case of the DHH participants, video (Sony, Japan, HDR-CX405) and audio (Olympus LS-P1) recordings of the replies of the participant and the sign language interpreter were made and subsequently transcribed by the researcher and interpreter.

The examiner started the testing procedure by introducing the participants to the task through a general instruction in which the way of questioning and answering was discussed. Thereafter, the participants were given a practice item to familiarize them with the task.

### Data Preparation and Data Analyses

#### Theory of Mind Construct

To investigate whether ToMotion assesses the unidimensional construct of ToM, a confirmatory factor analysis with one factor was conducted on the 12 ToM items using Maximum Likelihood extraction. Fit of the factor model was evaluated based on both the overall chi-square test and the root mean square error of approximation (RMSEA; Steiger, 1990; Browne and Cudeck, 1992) as a goodness-of-fit index. According to Brown and Cudeck, an RMSEA equal to or smaller than 0.05 can be considered a close fit, whereas an RMSEA larger than 0.1 is indicative of a poor fit.

Next, internal consistency reliability was calculated for the total score by calculating Cronbach’s alpha across the 12 ToM items. Following the discussion on the interpretation of Cronbach’s alpha values by Taber (2018), we considered Cronbach’s alpha’s values between 0.50 and 0.60 to indicate moderate reliability, values between 0.60 and 0.75 to indicate acceptable reliability, and values higher than 0.75 to indicate good reliability.

#### Group Comparisons on Theory of Mind Total

To investigate whether there were differences in the total ToM score between groups (TD/DHH/DLD), an ANOVA with a ToM total score as dependent variable and group (TD/DLD/DHH) as a between-subject factor was carried out. Age at testing was added as a covariate to control for age differences between groups.

#### Theory of Mind Sequence

To examine whether the ToMotion is able to measure the sequence of ToM in everyday situations, it was investigated whether there were differences in mean TOM question score between the descriptive/explanatory/personalized questions. A repeated measure ANOVA was done with the type of question (descriptive/explanatory/personalized) as within-subject factor and the mean ToM question score as the dependent variable. In addition, to investigate whether the groups (TD/DHH/DLD) differed on consistency patterns, a generalized linear model using a binominal distribution and logit link function was performed, with consistency score as the dependent variable and group (TD/DHH/DLD) as a between-subject factor. In both analyses, age at testing was added as a covariate to control for age differences between groups.

Data was analyzed using Statistical Package for the Social Sciences (SPSS) Statistics for Windows, Version 25.0.

## Results

### Measurement of the Theory of Mind Construct

One of the objectives of this study was to investigate whether ToMotion does measure one construct. The confirmatory factor analysis on the 12 ToM items revealed a good fit of the 1-factor model [*X*^2^ (54) = 55.379, *p* = 0.422, RMSEA = 0.02]. All factor loadings were positive, with 11 items having a factor loading higher than 0.30.

The reliability analysis of the ToM total score on the 12 ToM item scores revealed a Cronbach’s alpha of 0.76, suggesting that the test has good internal reliability.

### Typical vs. Atypical Groups on Theory of Mind Total

We hypothesized that both the DHH adolescents and the adolescents with DLD have lower ToM performance than their TD peers on the ToMotion task.

The ANOVA with ToM total score as dependent variable, group (TD/DLD/DHH) as between-subject factor, and controlled for age as covariate showed that the effect of group on ToM total score is significant and strong [*F*(2, 57) = 5.974, *p* = 0.004, *partial eta^2^* = 0.173]. Pairwise comparisons showed that, on average, the TD group has a significantly higher ToM total score (*m* = 52.2) than the DHH group (*m* = 42.0, *p* = 0.001), but the TD group does not have a significant higher ToM total score than the DLD group (*m* = 49.3, *p* = 0.340). The DLD group has, on average, a significantly higher ToM total score than the DHH group (*p* = 0.022) (see Table 2).

**TABLE 2:** Mean ToM total scores (standard errors within brackets), mean consistency scores, and standard errors within brackets for children in the TD-group, children in the DLD group, and children in the DHH group.

Mean (SE)	TD group	DLD group	DHH
ToMtotal score	52.2 (1.6)	49.3 (2.4)	42.0 (2.4)
Consistency score	0.73 (0.024)	0.67 (0.04)	0.77 (0.035)

### Theory of Mind Sequence

Similar to the ToM sequence in daily-life situations, we were interested in the performance of the participants on the questions that reflect the ToM sequence within the items, and if the participants are able to persist in this sequence across all items. We were also interested if there are differences between the typical and atypical groups concerning this sequence consistency. From a developmental perspective, we hypothesized that question 2 is more difficult than question 1, and that question 3 is more difficult than question 2. Furthermore, we hypothesized that the typical groups show more consistency in the ToM sequence in combination with a higher ToM score than the atypical groups.

The effect of question type (descriptive/explanatory/personalized) on mean ToM question total score was significant and strong [*F*(2, 59) = 38.086, *p* < 0.001, partial eta2 = 0.564]. Pairwise comparisons showed that the mean score on the descriptive question (*m* = 18.3) was significantly higher than the mean score on the explanatory question (*m* = 15.2, *p* < 0.001) and the mean score on the personalized question (*m* = 15.7, *p* < 0.001). The mean score on the explanatory question and the mean score on the personalized question did not significantly differ from each other (*p* = 0.120).

The analysis on the consistency scores showed that the effect of group (TD/DLD/DHH) on consistency score is not significant [*X*^2^ (2) = 3.872, *p* = 0.144], indicating that the groups do not differ significantly from each other with respect to mean consistency score. The DLD group (*m* = 0.67) scored lower than the TD group (*m* = 0.73), and the DHH group (*m* = 0.77) scored higher than both groups (see Table 2).

## Discussion

To address the need for an ecologically valid test instrument to assess ToM in Dutch adolescents with and without language and communication problems, the ToMotion has been constructed. An attempt was made to gain more insight into ToM functioning in adolescence from an ecological perspective.

The first question we were interested in was: do the 12 items together reflect a single construct?

From the results of the confirmatory factor analysis, we can conclude that the 12 different social situations we have created are able to measure 1 construct. This unidimensionality is assumed to reflect cognitive ToM, since the items predominantly involve thinking about the thoughts, knowledge, beliefs, and the intentions of others, which is characteristic for the sub-component cognitive ToM (Westby and Robinson, 2014). Of course, this is not meant to say that ToMotion cannot evoke somewhat more affective responses, especially since, in daily life, cognitive and affective components of ToM often interact (Schurz et al., 2020), but the extent of these responses is fairly limited.

The second research question we were interested in was: do the atypical groups differ in their performance on the ToMotion compared to typically developing peers?

Concerning the ToM total scores, TD adolescents significantly outperformed the DHH adolescents on ToM total score, as expected. Differences in performance (ToM total scores) between DLD and TD do not reach statistical significance, although the average scores point in the direction of TD performing better than DLD.

The third research question: does the ToMotion map a developmental sequence in ToM performance as exemplified by increasing difficulty of the questions asked in every item? In addition, are there differences in this sequence, both in terms of order as in terms of consistency between typical and atypical groups?

It was expected that there was a build-up in the difficulty of the questions. Particularly, from the joint attention (question 1) (Tomasello, 1995; Westby and Robinson, 2014) to the first-order ToM at 4–5 years of age (question 2) where the child can form a simple mental representation of what another thinks or feels (Michalson and Lewis, 1985; Pons et al., 2004). This is extended to the second order ToM at 6–12 years of age where the ability to predict what someone else thinks or feels emerges, along with the ability to determine what the other person thinks of how someone else feels or thinks and the development of an appropriate response (question 3) (Miller, 2009; Im-Bolter et al., 2016). All participants scored, on average, significantly higher on the first question than on questions 2 and 3, as expected. However, there was no significant difference found between questions 2 and 3. Moreover, there was no significant effect for differences in consistency scores between the DHH group and the TD or DLD group. Since the question type does not determine the difficulty of the social situation, the social situation as a whole determines the difficulty of the item. However, there does seem to be a trend in the average scores, pointing out that the DHH group scores are higher than DLD and TD on consistency scores.

## Conclusion

In conclusion, assessing ToM with the ToMotion results in a picture that DHH adolescents score lower than TD peers. However, their scores are as consistent as those of the TD peers. The difference cannot be attributed to less accessibility of the test since it was conducted in SLN. Our results are in line with the few studies performed in DHH (Lecciso et al., 2016; Marschark et al., 2018; de Gracia et al., 2020) adolescents. The apparent delay in ToM in DHH adolescents is most probably caused by language delays, not only in spoken language as a consequence of their hearing loss, but also in SLN, given the fact that most DHH adolescents have hearing parents. Consequently, sign language input was delayed until the time when parents learned sign language and/or when the DHH adolescents entered bilingual deaf education (Hermans et al., 2010). This backlog in language proficiency can cause a delay in the development of ToM from childhood. In the current sample, there is a relatively large number of deaf adolescents with deaf parents, which may give a distorted picture of the ToM backlogs seen in deaf participants with hearing parents.

The picture for DLD adolescents is the reverse. Quite surprisingly, these adolescents show no differences in ToM scores, but the scores seem to be somewhat more inconsistent compared to TD peers. Lancaster and Camarata (2019) proposed to view DLD, akin to autism spectrum disorder (ASD), as a spectrum disorder across severity levels rather than as a set of distinct subtypes. It may be that the relatively small sample in our study includes relatively language proficient adolescents with DLD. Unfortunately, we do not have data on language proficiency. Another explanation might be that the adolescents experienced ToM deficits in childhood that they caught up with in adolescence. On the other hand, this does not seem very probable since the only longitudinal studies show that ToM deficits in DLD persist in adult life (Clegg et al., 2005). In the future, more longitudinal research should be done on ToM development over the human lifespan.

Despite strengths of the study, there are some limitations to be mentioned. A larger sample size would be eligible in future work. Because the DHH adolescents are not present in large numbers in the entire population, there are some difficulties to find a large group that is also open to participation in test research. International cooperation could offer a solution. Also, a larger sample with better power is required to accommodate the wide variety of DLD.

To investigate the possible explanations for proficiency in ToM in DHH adolescents and adolescents with DLD, future research must include both assessment of language skills and tasks regarding social insight.

## Data Availability Statement

The raw data supporting the conclusions of this article will be made available by the authors, without undue reservation.

## Ethics Statement

The studies involving human participants were reviewed and approved by the ESCW. Written informed consent to participate in this study was provided by the participants’ legal guardian/next of kin. Written informed consent was obtained from the individual(s) for the publication of any potentially identifiable images or data included in this article.

## Author Contributions

LS, HK, LV, and CV contributed to the conception and design of the study. LS and IR-K collected, scored, and analyzed the data. LS prepared the first draft of the manuscript. All authors contributed to manuscript revision, read, and approved the submitted version.

## Conflict of Interest

The authors declare that the research was conducted in the absence of any commercial or financial relationships that could be construed as a potential conflict of interest.

## Publisher’s Note

All claims expressed in this article are solely those of the authors and do not necessarily represent those of their affiliated organizations, or those of the publisher, the editors and the reviewers. Any product that may be evaluated in this article, or claim that may be made by its manufacturer, is not guaranteed or endorsed by the publisher.
