# Supplementary material for: Measuring Theory of Mind in Adolescents With Language and Communication Problems: An Ecological Perspective
Source: Front Psychol. 2022 Apr 25;13:761434. doi: 10.3389/fpsyg.2022.761434 (PMC9081804; doi:10.3389/fpsyg.2022.761434)
Supplement: Supplementary file 1 [file Data_Sheet_1.pdf]

**Appendix 1: Scripts ToMotion**

All scenes were filmed in 1 take and you see both people from the front. All clips have a duration of approximately 20 seconds. The setting of the clips is continuous in the same school environment. It changes from canteen, to auditorium, to the space near the lockers.

| | |
|---|---|
| Item 1: Lie<br>*Amy and Lucas are in the school cafeteria. They both have a can of coke and go to the checkout.* | Amy: It's our turn to pay.<br>*Stows money in purse without Lucas noticing*<br>Amy: I forgot my money<br>Lucas: Okay, I'll pay. |
| Item 2: White Lie<br>*Amy and Lucas are sitting at a table. Lucas is drawing. Amy is reading.* | Lucas: I have almost finished my drawing, I am really proud of it.<br>*Show drawing of very simple doll. It is clear that this is not the level that someone his age should be able to draw.*<br>Lucas: What do you think?<br>Amy: Nice ! |
| Item 3: Joke<br>*Amy and Lucas are both sitting at the table in the cafeteria.* | Lucas: Tomorrow I'm going to play tennis with Pim.<br>Amy: Why not with a racket? |
| Item 4: Pretend<br>*Amy laughs and checks her phone. She has books in front of her. Lucas walks up. When Lucas arrives she quickly puts her phone away.* | Lucas: Hey Amy, what are you doing?<br>*Amy puts away her phone, tries to hide it from Lucas.*<br>Amy: Uhh my homework. |
| Item 5: Misunderstanding<br>*Amy is busy making a puzzle on the table. In her hand is one piece with the wrong side up. Lucas walks over to her.* | .<br>Amy: I am puzzling, but it is difficult.<br>Lucas: Turn around!<br>*Amy turns herself in stead of the piece of puzzle.* |

| | |
|---|---|
| Item 6: Persuasion<br><br>*Amy and Lucas sit opposite each other on a stone in a schoolyard. Behind them is a table tennis table. Lucas has two paddles in his hand.* | Amy: I would love to exercise more, but physical exercise is really not for me!<br>Lucas: Ah come on, go play table tennis with me.<br>*Lucas pushes a paddle into Amy's hands and touches her shoulders.*<br>Lucas: come on!<br>*Amy sighs and walks with Lucas to the tennis table.* |
| Item 7: Appearance/ reality<br><br>*Lucas has a box of a well-known brand of chocolates in his hands. He walks down a hall towards Amy.* | *Lucas holds a box of candy.*<br>Amy: Hey! Yummy! Can I have one too?<br>Lucas: Yes of course!<br>*Lucas opens the box, Amy looks in the box..there are nails in it ..*<br>Lucas laughs: yes I had to take it with me for the lessons manual labor. |
| Item 8: Idiom<br><br>*Lucas is doing his homework in the auditorium. Amy walks up to him.* | *Amy arrives and is visibly angry.*<br>Amy: I am really mad! I'm boiling with anger!<br>Lucas: Calm down, maybe you should cool off in the shower. |
| Item 9: Forgetting<br><br>*Amy and Lucas walk down the hall to the cafeteria, side by side.* | Lucas: I have to go to the toilet. Can you keep a spot for me?<br>Amy: I will!<br>*When he returns, Lucas sees that Amy is sitting with another girl, no spot left.*<br>Lucas: Hey Amy, wouldn't you keep a spot for me?<br>*Amy looks like she's forgotten.*<br>Amy: oh…. |
| Item 10: Double Bluff<br><br>*Lucas is sitting at the table in the cafeteria. Amy approaches from behind.* | *Lucas sends a lot of hearts to his girlfriend on WhatsApp with his phone. Amy comes up from behind and checks his phone.*<br>Amy: Hey! What are you doing.<br>*Lucas quickly puts his phone away.* |

| | |
|---|---|
| | Lucas: Texting heart emoji’s to my girlfriend ... all right ?! |
| Item 11: Contrary Emotions<br>*Lucas and Amy are in the school cafeteria.* | *Lucas and Amy are in the school canteen at the order counter.*<br>Lucas: From now on I will only eat healthy food!<br>*Lucas sees fruit and unhealthy snakcs. He looks at both.*<br>Lucas: Uh, 2 croquettes please. |
| Item 12: Irony<br>*Amy stands in front of the coffee machine. Lucas approaches from behind.* | *Amy's at the coffee machine. She removes the cup from under the machine too early, spilling everything over it.*<br>Lucas: Nice work !! |
| Practice Clip/ Lie | *Amy and Lucas read books. Lucas takes a packet of gum and takes one out. The package is still full and he puts it behind the stack of books. Amy asks if she can also have a chewing gum, she cannot see the package. Lucas says: No sorry, empty.* |

## Appendix 2 example scoring Item 8, Idiom

**Question 1**

| | |
|---|---|
| **2 points** | Core:<br>Lucas is sitting at the table when Amy approaches. Amy is angry and shows this emotion, boiling with anger. Lucas says take a shower then cool down (so to speak). |
| **1 point** | Part correct, contains both core elements:<br>• Amy shows anger, Lucas is working at the table. Amy says she is angry.<br>• Lucas tells Amy to shower.<br>Note: If the answer shows that Lucas says that she has to take a shower to cool down, the 2-point answer applies. |
| **0 points** | None of the above |

**Question 2**

| | |
|---|---|
| **2 points** | Reference to the fact that he wants to calm Amy through imagery. He means he sees that she's hot tempered / hot with anger / she looks hot with anger / so she needs to cool down; in a manner of speaking in the shower, but not literally. He means it lightly / as a joke with the aim of making her relax. |
| **1 point** | Partially correct e.g.<br>- She looks hot / overheated |
| **0 points** | It is assumed that there are incorrect intentions, for example: Lucas thinks you only get less angry when you shower, Amy smells, etc. |

**Question 3**

| | |
|---|---|
| **2 points** | Answer showing that she understands that Lucas does not really mean that she should take a shower, but that it is intended as a metaphor. Recognize imagery.<br>- Kidding back<br>- Relax |
| **1 point** | Minimal response that is still socially appropriate: e.g .:<br>- Yes, that's an idea.<br>- No, I remain angry |

| | |
|---|---|
| | - Continuing to ask what he means. |
| **0 points** | Not knowing, inappropriate for the social context e.g. thinking Lucas is making fun of you, has bad intentions, really taking a shower because Lucas says so. |

## Appendix 3 Consistency Scores and frequency

| Pattern | Consistency | #item1 | #item2 | #item3 | #item4 | #item5 | #item6 | #item7 | #item8 | #item9 | #item10 |
|---|---|---|---|---|---|---|---|---|---|---|---|
| 000 | C | 1 | | 3 | | 2 | | | 3 | 5 | 2 |
| 001 | I | 1 | 1 | | 2 | | | | 1 | 1 | |
| 002 | I | | | | | | | | | 1 | |
| 010 | I | | | 1 | 1 | | | | 1 | | 1 |
| 011 | I | | 1 | 1 | | | 1 | 1 | | | 1 |
| *012* | I | | | | | | | | | | |
| 020 | I | | | 1 | | | | | | | |
| *021* | I | | | | | | | | | | |
| *022* | I | | | | | | | | | | |
| | | | | | | | | | | | |
| 100 | C | | 1 | 4 | 1 | 2 | | | 1 | 1 | 4 |
| 101 | I | | 1 | 1 | 1 | | 1 | | 2 | 1 | 5 |
| 102 | I | | 1 | | | | | | | | |
| 110 | C | 1 | | | | | 1 | 4 | 2 | | 5 |
| 111 | C | 7 | 7 | 6 | 6 | 5 | 7 | 7 | 15 | 11 | 24 |
| 112 | I | 3 | 5 | 3 | 3 | 5 | 1 | 4 | 6 | 6 | 2 |
| 120 | I | | 1 | | | | | | | 1 | |
| 121 | I | 1 | 3 | 1 | | 1 | 4 | 3 | 1 | 1 | 2 |
| 122 | I | 2 | 4 | 4 | | 1 | | 1 | | 2 | |
| | | | | | | | | | | | |
| 200 | C | | 1 | 2 | 1 | 2 | 1 | | 2 | | 2 |
| 201 | I | 1 | | | | 1 | | | | 3 | |
| 202 | I | | 1 | | | 2 | | 1 | | 2 | |
| 210 | C | | | 1 | 1 | | | 3 | | | |
| 211 | C | 20 | | 1 | 6 | 5 | 11 | 12 | 8 | 6 | 7 |
| 212 | I | 10 | 3 | | 11 | 7 | 1 | 8 | 3 | 4 | 2 |
| 220 | C | | | 1 | | | 1 | | 1 | | |
| 221 | C | 1 | 12 | 5 | 3 | 5 | 12 | 5 | 2 | 2 | 2 |
| 222 | C | 9 | 18 | 25 | 21 | 22 | 20 | 12 | 9 | 14 | 2 |
| | | | | | | | | | | | |
